# Real-time monitoring and just-in-time intervention for adherence to pre-exposure prophylaxis among men who have sex with men in China: a multicentre RCT study protocol

**DOI:** 10.1186/s12889-020-08709-2

**Published:** 2020-07-24

**Authors:** Xia Jin, Hongyi Wang, Hang Li, Zhenxing Chu, Jing Zhang, Qinghai Hu, Wei Lv, Xiaojie Huang, Yaokai Chen, Hui Wang, Xiaoqing He, Lukun Zhang, Zhili Hu, Rantong Bao, Shangcao Li, Haibo Ding, Wenqing Geng, Yongjun Jiang, Hong Shang, Junjie Xu, for the CROPrEP Study Team, Xiaoyun Shi, Rui Li, Yangyang Gao, Yanni Ma, Yi Duan, Guanghui Zhang, Yao Li, Fang Zhao

**Affiliations:** 1 NHC Key Laboratory of AIDS Immunology (China Medical University), National Clinical Research Center for Laboratory Medicine, The First Affiliated Hospital of China Medical University, Shenyang, 110001 China; 2 Key Laboratory of AIDS Immunology, Chinese Academy of Medical Sciences, Shenyang, 110001 China; 3 Key Laboratory of AIDS Immunology of Liaoning Province, Shenyang, 110001 China; 4 Collaborative Innovation Center for Diagnosis and Treatment of Infectious Diseases, 79 Qingchun Street, Hangzhou, 310003 China; 5 Peking Union Medical College Hospital, Chinese Academy of Medical Sciences, Beijing, 100730 China; 6 Beijing Youan Hospital, Capital Medical University, Beijing, 100069 China; 7 Chongqing Public Health Medical Center, Chongqing, 400036 China; 8 Shenzhen Third People’s Hospital, Shenzhen, 518000 Guangdong China

**Keywords:** HIV, men who have sex with men, Medication adherence, Pre-exposure prophylaxis, Internet of things, Electronic drug monitors

## Abstract

**Background:**

Pre-exposure prophylaxis (PrEP) is an effective biomedical strategy to prevent transmission of HIV infection, although medication adherence remains a challenge. We present the protocol for a multicentre randomised controlled trial to measure the effectiveness of a real-time monitoring and just-in-time intervention on medication adherence among PrEP users in China.

**Methods:**

Study participants will include 1000 men who have sex with men (MSM) from four cites in China (Shenyang, Beijing, Chongqing and Shenzhen) attending a tenofovir disoproxil fumarate/emtricitabine (TDF/FTC) PrEP project as part of a real-world, prospective multicentre cohort study (*CROPrEP*). Participants will be randomised into the intervention and control arms in a 1:1 ratio. Participants in the intervention arm will be provided with remote real-time monitoring equipment that triggers twice just-in-time SMS (Short Messaging Service) medication reminders to PrEP users every half an hour when a scheduled dosage is missed, and followed with just-in-time SMS medication reminders to clinicians half an hour when there is no supplement after the second just-in-time SMS reminder to PrEP users. Clinicians will initiate individualised telephone intervention as soon as possible upon receipt of the just-in-time SMS missed dose alert. Those in the control arm will only receive generic weekly SMS reminders. The study will last 6 months. Participants will be seen at follow-up visits at three and 6 months. Trial outcomes to be measured include self-reported adherence assessed via questionnaire and pill counts, as well as drug concentration test results.

**Discussion:**

Medication adherence is critical to achieve optimal benefits from PrEP. This study will be the first individualised behaviour intervention using real-time technology to increase adherence among MSM PrEP users globally. If found effective, a real-time monitoring and just-in-time intervention system may be utilized for improving adherence and thus effectiveness of global PrEP application.

**Trial registration:**

This study registered at ClinicalTrials.gov (ChiCTR1900025604) on September 2, 2019.

## Background

Pre-exposure prophylaxis (PrEP) is recognized as highly effective in preventing new HIV infections in high-risk populations [1–9]. However, multiple clinical trials worldwide have shown that medication adherence to PrEP is suboptimal [6, 10, 11], which reduces its effectiveness. The literature on medication adherence interventions provides several examples in the field of antiretroviral therapy (ART) and other clinical areas [12–14]. However, while scientific consensus on the efficacy of PrEP has been attained, there has been little work in development and evaluation of interventions to improve PrEP medication adherence.

Measuring adherence to PrEP is challenging and has traditionally been done using self-report questionnaires and by measuring pill counts and blood drug concentrations [15]. All of these methods are subject to limitations posed by the intermittent nature of data collection. For example, missed doses may be detected several weeks to months after they occur. In addition, interventions can only be conducted after poor medication adherence is detected by clinicians through follow-up interviews, pill counts, drug concentration, and adherence scales. Therefore, it is often impossible to initiate medication adherence interventions before viral rebound that would lead to treatment failure and drug resistance.

Electronic drug monitors (EDM) constitute a novel technology that has been applied in clinical practice for real-time adherence detection, reminder of adherence lapses, and interventions to resume treatment before treatment failure and onset of drug resistance [16–18]. The EDM system involves the use of a microchip embedded inside an intelligent pill container that records when the container is opened as an indication that the medication has been taken. A key innovation of advanced EDM technologies is to combine the Internet of Things with smartphone technology to monitor adherence in real time and provide personalised intervention. Patients can set their medication reminders (pillbox ringing and mobile phone reminders) to notify them when a scheduled dose is missed. The EDM system stores and uploads medication use data to support joint management by clinicians, the patient, and family members to improve medication adherence. Graphical adherence curves and scoring information can be generated on linked mobile phones, supporting real-time intervention from professionals when needed to avoid missed doses and overdoses.

This technology has been used to improve medication adherence of HIV treatment and other chronic diseases. However, the device has not yet been tested for PrEP. We now present a study protocol examining the use of EDM technology to monitor PrEP adherence in real time, along with personalised intervention to users to assess improvements in medication adherence with this system. This randomised controlled trial will prospectively assess the efficacy of this approach, providing scientific evidence to guide development of future PrEP intervention strategies.

## Methods

### Objectives

The objective of the study is to estimate the effectiveness of real-time monitoring and just-in-time intervention technology for improving PrEP medication adherence among Chinese MSM.

### Study setting

The study includes four study centres (First Affiliated Hospital of China Medical University in Shenyang, Beijing You’An Hospital of Capital Medical University in Beijing, Shenzhen Third People’s Hospital in Shenzhen, and Chongqing Public Health Medical Treatment Centre in Chongqing).

### Trial design

This study is organized within the ongoing *CROPrEP* project, a multicentre, real-world prospective cohort study of daily or event-driven PrEP (emtricitabine/tenofovir disoproxil fumarate, TDF/FTC) among 1000 HIV-negative MSM most-at-risk of HIV infection in China. For the present study protocol, we randomly divided the 1000 *CROPrEP* study participants (500 from daily TDF/FTC group and 500 from event-driven TDF/FTC group in the *CROPrEP* study) into intervention and control arms in a 1:1 ratio (250 in intervention arm and 250 in control arm for each of the original *CROPrEP* study groups).

### Eligibility criteria

The current RCT will be conducted with participants in the ongoing *CROPrEP* study. The inclusion criteria for the *CROPrEP* study include: (1) HIV-negative MSM; (2) aged 18–65 years; (3) report no severe damage to liver or kidney function; (4) self-report at least one of the following behaviours during the last 6 months: engaging in unprotected receptive anal sex with men, multiple male sexual partners, current or previous STIs (syphilis, gonorrhoea, chlamydia, chancroid, venereal lymphogranuloma, or other STI), had a history of post-exposure prophylaxis (PEP) but did not receive PEP in the last month; (5) not planning to leave local area of residence within the next 6 months (to ensure cohort retention); and (6) being able and agreeing to provide written informed consent. Individuals who maintained a monogamous sexual relationship with only one HIV-1 negative partner or one virologically suppressed HIV-1 positive partner for more than 1 year will be excluded.

### Interventions

**Fig. 1:**
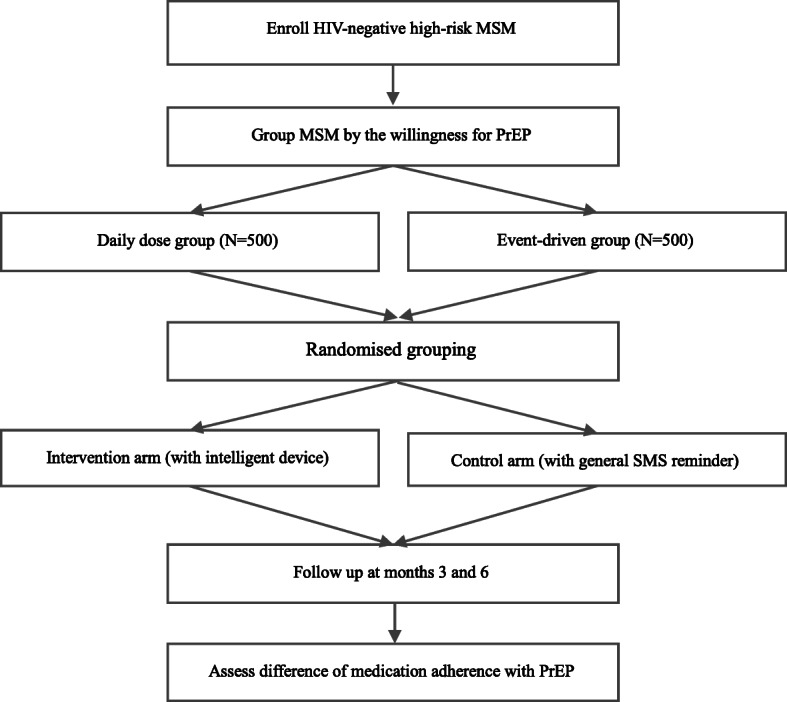
Study flow chart

Participants in the intervention arm will be provided with a real-time monitoring device administering twice just-in-time SMS medication reminders to PrEP users every half an hour when a scheduled dosage is missed and followed with just-in-time SMS medication reminders to clinicians half an hour when there is no supplement after the second just-in-time SMS reminder to PrEP users. Clinicians will initiate individualised telephone intervention as soon as possible when they receive the just-in-time SMS medication reminder. Telephone interventions will be carried out at the same day for reminders received before 4:00 p.m. and 8:00 a.m. the next day for reminders received after 4:00 p.m., whereas control arm participants will receive general weekly SMS medication reminders. Participants in both groups will be invited to attend follow-up clinic visits at three and 6 months. The flow chart depicting the study design is shown in Fig. 1.

### Sample size

We have powered the RCT to be able to detect meaningful changes in optimal adherence. Our estimates of outcome differences are based on our basic findings from our earlier pilot study with Chinese PrEP users, in which we found that proportions of adherence were 94% versus 86%, in intervention versus control patients after 6 months of the intervention. We will recruit 500 PrEP users per study arm. With the consideration of a two-sided alpha of 0.05 and 40% loss to follow-up of our study, we will have a 90.6% power at to detect specified differences in adherence between the two arms after intervention.

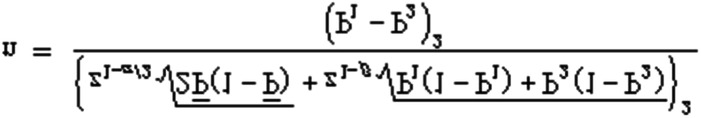



### Real-time monitoring equipment

**Fig. 2:**
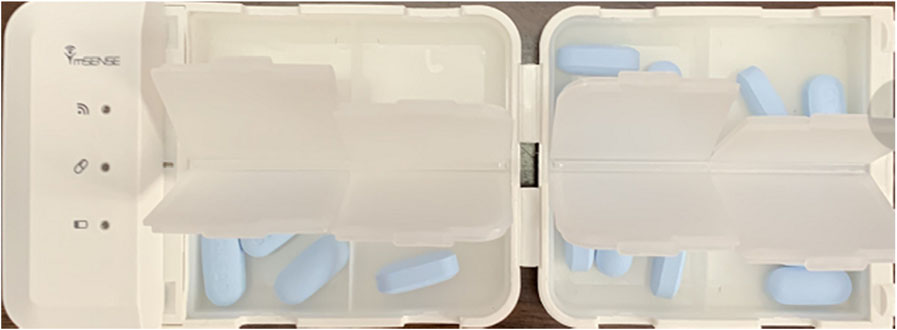
Real-time monitoring device (Icompanion container) Note: The device was designed by Msense, Icompanion Health Technologies, Guangzhou, China. This figure is the real scene shooting by our CROPrEP Study Team. It was not derived from any published source and the copyright permissions for reuse is belonged to CROPrEP Study Team

The medication adherence monitor (Msense, Icompanion Health Technologies, Guangzhou, China) records dosing behaviour in real time using microchips embedded in the container vial cap to detect when the container is opened (Fig. 2). This approach assumes that the participant takes the medication every time the monitored equipment is opened.

**Fig. 3:**
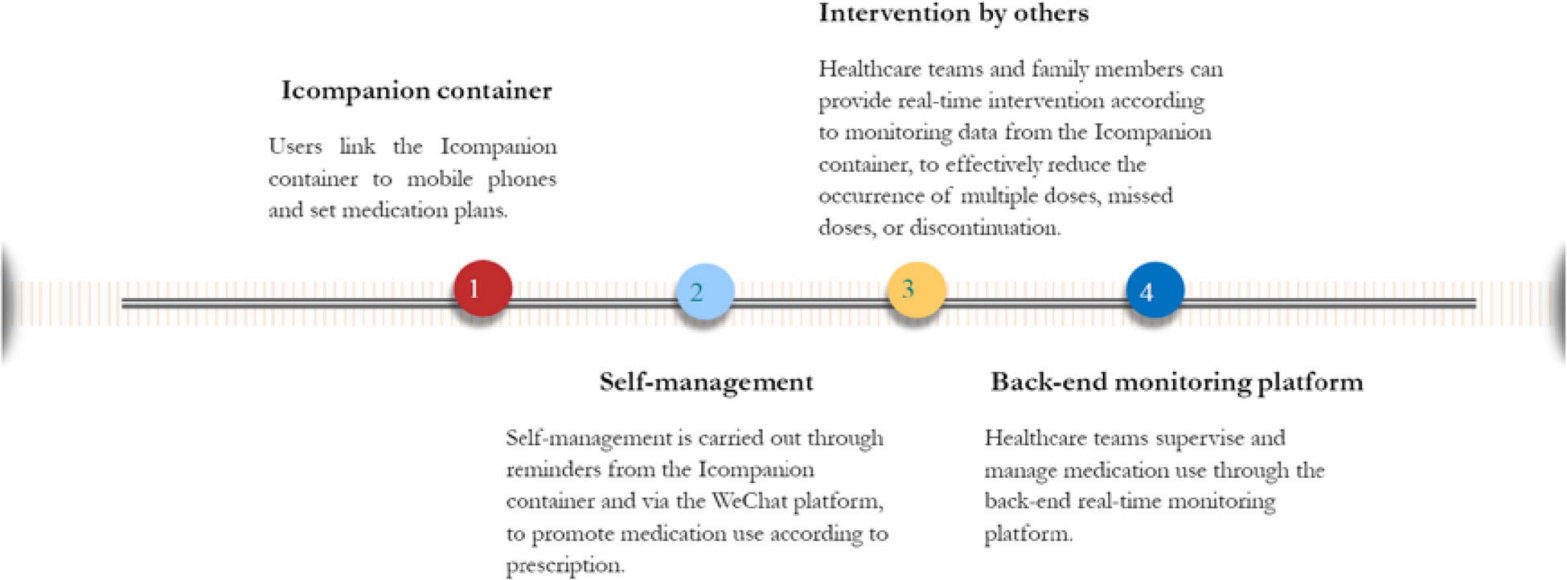
Intervention procedure using a real-time monitoring device

The real-time monitoring device can set the medication time, issue reminders for medicine to be taken, and trigger voice alerts for repeat doses. Data are automatically uploaded to a cloud-based server and can then be accessed by clinicians, PrEP users and family members via WeChat platform. Clinicians can monitor real-time data on PrEP use in the intervention arm from a back-end platform and can provide just-in-time intervention to participants who did not take medicine according to prescription. PrEP users can also check their records on medication taken and not taken, to monitor their own medication usage and for reference during clinic visits. Graphical medication adherence curves and scoring data can be generated on mobile phones to support self-management and personalised reminders. If participants in the intervention arm do not take medication on time according to prescription, clinicians and family members can send a message or initiate voice calls directly to the patient. Details on the intervention procedure are illustrated in Fig. 3.

### Randomisation

Following the baseline study visit, all participants are randomised to one of the two study arms using 1:1 randomisation. The PASS 11.0 randomisation module will be used to ensure equal sizes of the two study groups. Each group will have 500 participants, and two groups of 500 random numbers will be generated for randomised grouping of the daily dose group and the event-driven group to ensure equality of the two medication dosing strategies within the intervention arm (Table 1).

**Table 1:** Illustration of randomisation procedure

Serial number	Group^a^	Maximum deviation ratio to target (%)	Cumulative sample size of two groups (A, B)
1	B	0.1%	(0, 1)
2	A	0.0%	(1,1)
3	A	0.1%	(2, 1)
998	B	0.0%	(499, 498)
999	A	0.1%	(500, 499)
1000	B	0.0%	(500, 500)

^a^ A, intervention arm; B, control arm

Accordingly, both the PrEP daily FTC/TDF group and the event-driven FTC/TDF group will have 250 PrEP users randomly assigned to the intervention arm and 250 assigned to the control arm. Recruitment targets of four study sites were determined based on patient volume and are shown in Table 2.

**Table 2:** Number of participants to be recruited at each site

Sites	Intervention arm	Control arm
Shenyang	185	185
Beijing	210	210
Shenzhen	45	45
Chongqing	60	60
Total	500	500

All study participants will be seen for a baseline study visit, followed up at 3 and 6 months, and fill an online self-administered questionnaire assessing sexual behaviours, psychological status, self-reported medication adherence, attitude and side effects towards PrEP use. Blood samples and dry blood spots will be collected and tested as previously described for the *CROPrEP* project [19]. Pills remaining will be counted at the 3- and 6-month follow-up visits, and refills will be provided to last until the next study or clinic visit. Participants will be required to return the real-time monitoring device provided by the program when the study is completed.

The program will incorporate locally trained community-based organizations (CBOs) serving MSM to recruit eligible participants for the study. CBO peer educators will be responsible for guiding the correct use of the monitoring device and will provide support services through social media platforms (WeChat, QQ). The study also offers incentives of US $10 upon completion of the enrolment visit and US $10 each at the 3- and 6-month visits (US $30 total) to compensate the time contributed by participants.

### Laboratory procedure

The laboratory testing procedures and parameters are described in the published protocol of the *CROPrEP* project [19]. MSM who are enrolled in the study and later screen as HIV positive will be instructed to discontinue PrEP and visit the local study hospital for HIV confirmation.

### Data collection and management

The study questionnaire will be administered via a specialised online platform (Gold Data Technologies, Sichuan, China). Trained staff will collect data on remaining pill counts and verify test results.

CBO peer educators will instruct study participants on use of the real-time monitoring device at the baseline study visit and throughout follow-up using related images and videos in order to avoid overestimates (opening the container without removing pills) or underestimates (multiple pills removed during one opening of the monitoring device) of actual medication adherence. The monitoring device battery is sufficient to run in standby mode for 45 days after a 2-h charge.

### Study endpoints

Medication adherence refers to the degree to which a patient complies with the doctor’s prescription to complete the treatment plan. It involves taking medication regularly, not forgetting to take medication, and taking medication at the correct time, i.e., taking medicine according to the prescribed timing, dosage, and administration instructions. PrEP medication adherence will be measured via self-report questionnaires and pill counts as well as drug concentration test results. Details on the study endpoints are shown in Table 3.

**Table 3:** Study endpoints

**Medication adherence scores** - The proportion of self-reported number of pills taken among the number of pills prescribed by the doctor. - The proportion of the counted number of pills taken among the number of pills prescribed by the doctor. - The proportion of pills taken as calculated by remote real-time monitoring equipment among the number of prescribed pills by the doctor. Scores will be classified as high medication adherence (≥90%), intermediate medication adherence (≥60 and < 90%), or low medication adherence (< 60%).	
**Blood medicine concentration monitoring (TDF/FTC)** Blood TDF/FTC concentration will be classified as high medication adherence (≥90% concentration), intermediate medication adherence (≥60 and < 90%), or low medication adherence (< 60%).	

### Statistical methods

Normal distribution continuous variables will be presented by means and standard deviations while non-normal distribution continuous variables will be presented by medians and interquartile ranges. For categorical variables, composition ratios and 95% confidence intervals will be used to describe the variables. Chi-square analysis will be used to assess differences between the study arms in HIV testing history, HIV self-testing (HIVST) characteristics, and high-risk behaviours. Multinomial logistic regression will be used to measure the outcome of real-time monitoring equipment on medication adherence.

### Ethics and dissemination

Written informed consent will be provided to all study participants before take part in this study. The study was approved by the First Affiliated Hospital of China Medical University Ethics Review Committee ([2019]2019–253-3).

## Discussion

Traditional methods of measuring medication adherence such as self-report and pill counting are executed intermittently or at the end of the study period, often several months after the dosage has been missed. Accordingly, these methods often cannot guide intervention before the disease recurs or worsens. Hence, the ability to change medication adherence and prevent treatment failure or drug resistance with these methods is limited. In contrast, EDM is characterised by real-time monitoring and data processing, which allow healthcare teams and family members to monitor medication adherence and send reminders just-in-time to patients who have missed doses, before the disease may worsen. EDM is therefore a more efficient strategy to improve medication adherence.

Medication adherence is crucial for achieving optimal benefits from PrEP. However, many participants over-report PrEP medication adherence by traditional approaches such as self-reporting and pill counting, which adds to the challenge of accurately measuring medication adherence [15]. With traditional approaches, measurement of adherence after doses have been missed leads to an inability to provide timely intervention and thus to worse adherence. Real-time monitoring technology creates the ability to monitor medication adherence between routine visits and can trigger just-in-time intervention before adherence has lapsed for a substantial amount of time. Our study will be the first to directly compare traditional measurement of adherence versus real-time monitoring with just-in-time individualised intervention in PrEP users.

This study is focused on a novel, individualised behaviour intervention to reduce the risk of decreased medication adherence among PrEP users in China. Ours will be the first study of this type of intervention for improvement of PrEP medication adherence. Just-in-time telephone intervention requires investment of more time and energy. Our intervention employs novel technology combined with just-in-time SMS medication reminders and individualised telephone intervention to increase the intensity and efficiency of improving PrEP medication adherence in real time. Furthermore, telephone intervention only focuses on those with a missed dosage after just-in-time SMS reminders which can save time and energy. The control group will receive weekly universal undifferentiated SMS medication reminders since the real-time medication condition is unknown. Clinicians can provide just-in-time professional intervention based on information from the back-end real-time monitoring platform to improve the efficacy of PrEP in preventing the transmission of HIV infection. Dose-to-dose medication adherence data and curves generated on adherence patterns are informative in promoting self-management and intervention from others who are engaged with participants. The study has several important strengths, including capitalizing on the infrastructure of a large-scale, real-world multicentre study in China, use of real-time monitoring devices with just-in-time intervention to advance medication adherence, and measure the efficacy of such devices compared to traditional approaches.

Our study tests a real-time monitoring device with just-in-time intervention against other traditional approaches to address medication adherence in PrEP users. We hypothesize that by targeting real-time monitoring and intervention to reduce the risk of viral rebound, our strategy will improve TDF/FTC medication adherence, allowing clinicians and family members to maintain confidence regarding adherence.

Our study should be evaluated considering several limitations. First, the *CROPrEP* project is focused on the MSM population, in which medication adherence characteristics may be different from other patient populations. It has been shown that research study participants generally exhibit higher medication adherence levels than other clinical patients [20]. Accordingly, we recommend that future studies be conducted in other key populations to validate the effects of real-time monitoring and just-in-time intervention on PrEP medication adherence. In addition, the cost of real-time monitoring devices has limited their large-scale uptake in research and general clinical care. Thus, development of low-cost versions of these devices will be critically important for their future application.

## Data Availability

Not applicable.

## Abbreviations

PrEP: Pre-exposure prophylaxis
MSM: Men who have sex with men
TDF/FTC: Tenofovir disoproxil fumarate/emtricitabine
SMS: Short Messaging Service
ART: Antiretroviral therapy
EDM: Electronic drug monitors
HIVST: HIV self-testing
